# Repulsive guidance molecule A (RGMA) is widely expressed in the CNS of the sea lamprey *Petromyzon marinus* during early development.

**DOI:** 10.17912/micropub.biology.001145

**Published:** 2024-03-13

**Authors:** Laura González-Llera, Michael I. Shifman, Antón Barreiro-Iglesias

**Affiliations:** 1 Department of Functional Biology, University of Santiago de Compostela, Santiago de Compostela, Galicia, Spain; 2 Neuroscience Consulting, LLC.

## Abstract

RGM interactions with its receptor Neogenin play an important role in the regulation of axonal guidance or cell death in the developing central nervous system. The sea lamprey *RGMA* transcript has been recently identified. However, its expression has been only studied in the spinal cord of mature (premetamorphic) larval sea lampreys. Here, we report the expression of the sea lamprey *RGMA* transcript in developing embryos and prolarvae by means of in situ hybridization. Our data show that the *RGMA* transcript is broadly expressed in the central nervous system of embryos and prolarvae and with a rostro-caudal gradient of expression.

**Figure 1. Expression of RGMA , as determined by whole mount ISH, showed in sections of embryos and prolarvae of the sea lamprey:**
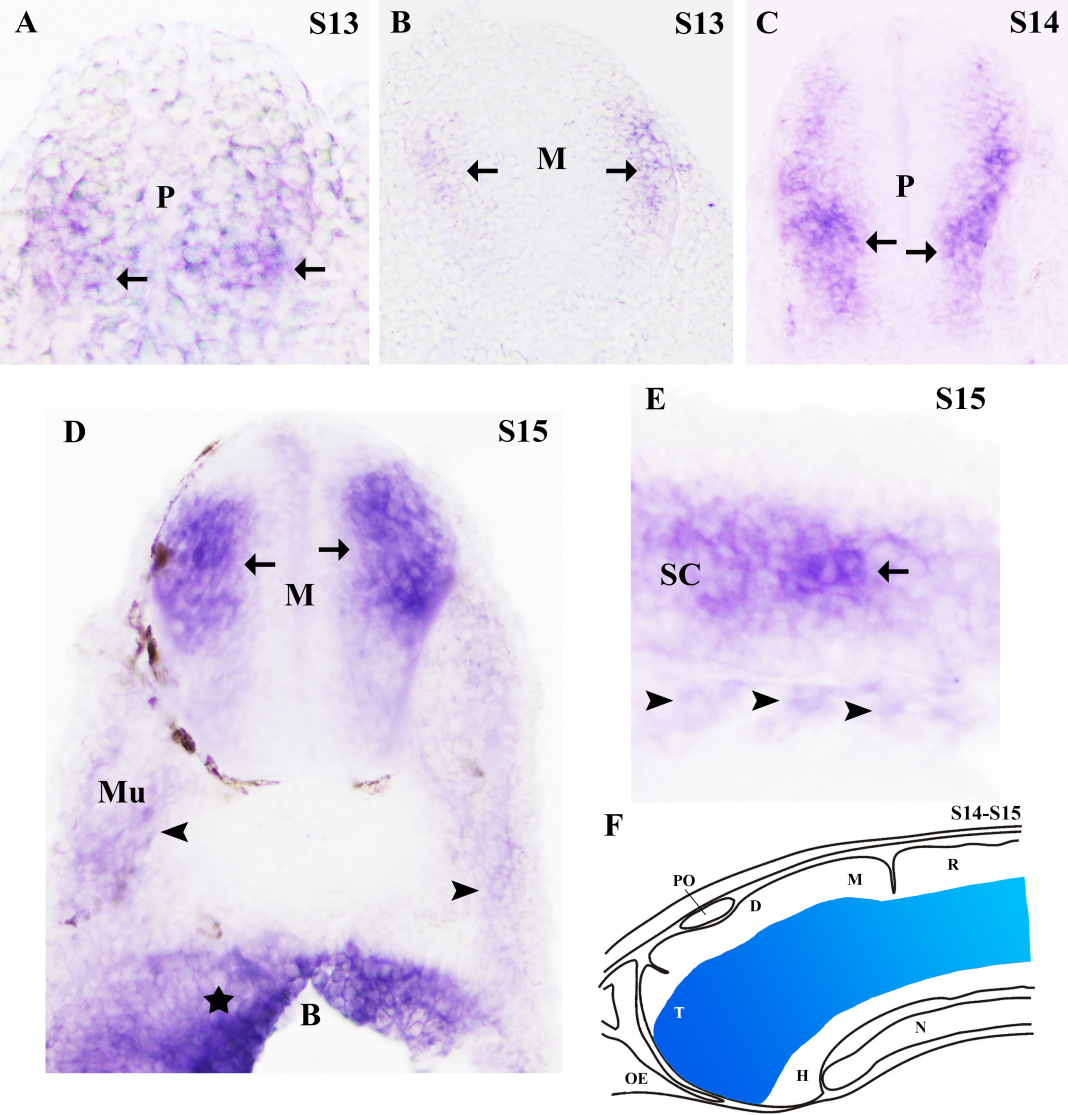
**A:** Photomicrograph of a transverse section of a S13 embryo showing *RGMA* transcript expression at ventral and medial levels of the rostral prosencephalon. **B:** Photomicrograph of a transverse section of a S13 embryo showing weak *RGMA* transcript expression at medial levels of the mesencephalon. Note the absence of labelling in the dorsal and ventral parts of the mesencephalon. **C:** Photomicrograph of a transverse section of a S14 prolarva showing *RGMA* transcript expression at medial and ventral levels of the prosencephalon. Note the absence of *RGMA* labelling in the dorsal margin of the brain. **D:** Photomicrograph of a transverse section of a S15 embryo showing *RGMA* transcript expression at medial levels of the mesencephalon. Note the presence of weaker *RGMA* labelling in the musculature (arrowheads) of the body walls and strong labelling in the branchial arch (star). Brown structures are some of the pigmented cells observed at this developmental stage. **E:** Photomicrograph of a sagittal section of a S15 prolarva showing weak *RGMA* transcript expression at medial levels of the spinal cord and in the somites of the musculature (arrowheads). Arrows point to CNS regions showing *RGMA* transcript expression. **F:** Schematic drawing of S14-S15 prolarvae summarizing the expression of the *RGMA* transcript in the brain at these developmental stages (indicated in blue). Note the absence of *RGMA* expression in periventricular regions of the neural tube in embryos and prolarvae (A-D) and its presence only in lateral margins of the neural tube (A-D). Abbreviations: B: branchial arch; D: diencephalon; H: hypothalamus; M: mesencephalon; Mu: musculature; N: notochord; OE: olfactory epithelium; P: prosencephalon; PO: pineal organ **;** R: rhombencephalon; SC: spinal cord; S13-15: Piavis’ developmental stages for *Petromyzon marinus* ; T: telencephalon.

## Description

Repulsive guidance molecule (RGM) is a glycoprotein with repulsive properties on growing axons that was first identified in chicks (Stahl et al., 1990) . In mammals, three homologues of the chick RGM (Stahl et al., 1990) have been identified: RGM A, B (also known as DRAGON) and C (also known as Hemojuvelin) (Schmidtmer and Engelkamp, 2004; Oldekamp et al., 2004; Niederkofler et al. 2004; Rajagopalan et al. 2004) . In mammals, RGMA and B are expressed in the nervous system, whereas RGMC is expressed in the muscles at early developmental stages (Schmidtmer and Engelkamp, 2004; Oldekamp et al., 2004) . In *Xenopus laevis RGMA* is also expressed in the developing central nervous system (CNS) (Gessert et al., 2008) . RGMs bind the type 1 transmembrane protein Neogenin and many of the reported biological effects of RGMs, such as roles on axon guidance or neuronal survival, rely on its interaction with Neogenin receptors (Rajagopalan et al. 2004; Matsunaga et al., 2004; see Siebold et al., 2017) . In the developing CNS, RGM-Neogenin interactions regulate axon guidance, establishment of the pseudostratified epithelium of the neural tube or neuronal differentiation (see De Vries and Cooper, 2008). Recently, an *RGM* ( *RGMA* ) gene/transcript has been identified in the sea lamprey (Shifman et al., 2009) . Lampreys belong to the oldest group of extant vertebrates, the agnathans or jawless vertebrates. Thus, the study of RGMA in lampreys can provide valuable information on the evolution of this signalling system in vertebrates. Moreover, lampreys are also a model of interest for the study of spontaneous axon regeneration after spinal cord injury (SCI) (e.g., González-Llera et al., 2022, 2024). Recent work revealed that Neogenin inhibition promotes axon regeneration and neuronal survival in lampreys after a complete SCI (Chen and Shifman, 2019) . Expression of Neogenin has been studied in giant descending neurons of the brainstem by in situ hybridization (ISH) (Shifman et al., 2009; Barreiro-Iglesias et al., 2014; Chen et al., 2017; Chen and Shifman, 2019) and by immunofluorescence in the spinal cord (González-Llera et al., 2023) of mature larval sea lampreys. *RGMA* expression has been studied only in the spinal cord of mature larval sea lampreys by means of ISH (Shifman et al., 2009) . Thus, data on the expression of RGMA in the CNS of early developing lampreys is lacking. Here, we report the expression of the *RGMA* transcript in developing sea lamprey embryos and prolarvae by using ISH. For this, we generated a riboprobe against a 581-nucleotide sequence of the sea lamprey ( *Petromyzon marinus* ) *RGMA* mRNA (see Methods section).

In pre-hatching embryos [stage 13 (S13)], the neural tube was still a solid neuroepithelial rod and the boundaries between the primary brain vesicles were hardly appreciable. The prosencephalic and mesencephalic regions consist of a thick ventricular zone formed by neuroepithelial cells and a primordial marginal zone formed by processes of these neuroepithelial cells. The rhombencephalon and spinal cord are cylindrical with little variation in diameter, exhibiting only a ventricular zone on both sides of a vertical slit-shaped cavity. In prolarvae (S14 and S15), two transverse ventricular sulci, the anterior and posterior intraencephalic sulci separate the diencephalic/mesencephalic primordium from the primordia of the telencephalon and rhombencephalon, respectively. The first neuronal populations become recognizable during late embryonic and early prolarval periods (Villar-Cheda et al., 2006; Barreiro-Iglesias et al., 2008a).

The expression pattern of the *RGMA* transcripts was studied by whole mount ISH in embryos and prolarvae between stages 13 to 15. The presence of *RGMA* transcript expression in peripheral tissues impeded to obtain high-quality photomicrographs of whole mounted samples; therefore, they were sectioned in transverse or sagittal planes after whole mount ISH for the better observation of the pattern of *RGMA* transcript expression in the CNS. In embryos (S13), *RGMA* transcript expression was observed in the neural tube, in addition to a weak labelling observed in the somites of the body wall musculature (Figs. 1A and B). *RGMA* expression was observed along the whole neural tube showing a rostro-caudal gradient of expression, whit the strongest expression in the prosencephalon ( Fig. 1A ) and the weakest in the caudal rhombencephalon/spinal cord. *RGMA* transcript expression was observed in lateral regions of the neural tube and not in periventricular locations. In the prosencephalon *RGMA* expression was observed in both the medial and ventral margins ( Fig. 1A ), whereas it was never present in dorsal most regions. Caudally, in the mesencephalon ( Fig. 1B ), rhombencephalon and spinal cord, *RGMA* expression was only observed in latero-medial margins of the neural tube, being the dorsal and ventral parts of the neural tube devoid of *RGMA* transcript expression at these CNS levels. From hatching onwards (i.e., prolarval stages S14 and S15), *RGMA* transcript expression was also observed in neural crest derived structures of the branchial arches ( Fig. 1 D ). The branchial arches are recognizable for the first time in 1-day post-hatching prolarvae. Faint *RGMA* transcript expression was also observed in the musculature of prolarval samples (Figs. 1C, D and E). Although the intensity of *RGMA* expression was higher in prolarvae than in embryos ( Fig. 1 ), no appreciable differences were observed in the spatial distribution of the *RGMA* transcripts in the CNS ( Fig. 1F ). In prolarvae, a rostro-caudal gradient of *RGMA* expression was observed as in embryos, with higher degree of expression in the prosencephalon that was weaker caudally ( Fig. 1F ). In prolarvae, as observed in embryos, *RGMA* expression was present in the ventral and medial portions of the prosencephalon (Figs. 1C and F). In the mesencephalon/rhombencephalon/spinal cord, *RGMA* transcript expression was only observed at medial levels (Figs. 1D, E and F) and both the dorsal and ventral most parts lacked *RGMA* expression (Figs. 1D and F).

Our ISH data show that *RGMA* is broadly expressed in the CNS at early embryonic and prolarval stages in lampreys, which suggest its possible involvement in the establishment of the first axonal pathways and differentiation of the first neuronal populations. The expression pattern of *RGMA* in the sea lamprey revealed similarities (and a few differences) with the expression pattern of RGMA2 in *Xenopus* embryos (Gessert et al., 2008) . In both sea lamprey and *Xenopus* , RGMA/RGMA2 are expressed in the whole neural tube with strongest expression in the prosencephalon. However, in *Xenopus* , RGMA2 is expressed in both medial and ventral portions of the whole neural tube (Gessert et al., 2008) , whereas in the sea lamprey this pattern of expression is only observed in the prosencephalon. In chick embryos (E9.5 and E10.5), RGMA is expressed in the ventral neural tube (Schmidtmer and Engelkamp, 2004) . In both, sea lamprey and *Xenopus* , *RGMA/RGMA2* transcripts are expressed in the developing branchial archs and muscles, which indicates a conserved role for RGMA in the development of these structures in anamniotes (agnathans and amphibians). In mouse, RGMA is also expressed in the retina at early post-natal days (P0). In our preparations no *RGMA* transcript expression was detected in the prolarval retina, this difference between mammals and lampreys might be related to the unusual development of the visual system in lampreys, which is delayed in comparison to other vertebrates since it does not finish until the metamorphosis (Meléndez-Ferro et al., 2002; Villar-Cheda et al., 2008; Cornide-Petronio et al., 2011).

In sea lamprey embryos and prolarvae, *RGMA* transcript expression was observed in a rostro-caudal column located at ventro-medial levels in the prosencephalon and in medial levels in the other regions, as observed in transverse sections. In the sea lamprey, the medial and dorsolateral longitudinal fascicles are the first longitudinal axonal bundles observed to develop in the neural tube, followed by the tract of the postoptic commissure and the supraoptic tract (Barreiro-Iglesias et al., 2008a). In lampreys, establishment of the first dorso-ventral tracts occurs after the appearance of the tract of the postoptic commissure and the medial longitudinal fascicle, the basal plate longitudinal axonal system (Barreiro-Iglesias et al., 2008a). Present results show that *RGMA* is expressed dorsally to the region where the first rostro-caudal axonal system (medial longitudinal fascicle) navigates at early developmental stages. So, RGMA could be involved in the establishment of the early scaffold of axon tracts by generating an inhibitory region for axonal growth. A chemorepulsive response arising from the interaction between RGMA and Neogenin has been shown for axons navigating along the supraoptic tract of *Xenopus* embryos (Wilson and Key; 2006) . Present results suggest a similar situation in lampreys but if a Neogenin-RGMA interaction applies for lamprey early axonal tracts should be tested in further studies showing which early neuronal populations of lampreys express Neogenin and by functional manipulations of this signalling system.

## Methods

*Animals*

Pre-hatching embryos (stage 13, n=8), hatchlings (stage 14, n = 8) and prolarvae in pigmetation stage (stage 15, n = 14) of sea lamprey ( *Petromyzon marinus* L.) were used. Adult male and female lampreys were collected in the River Ulla (Galicia, Spain) during the breeding season. The eggs were laid and fertilized in the laboratory and kept in fresh oxygenated water at 15°C, until desired stages were obtained. Embryos and prolarvae were staged according to the table established by Piavis (1971) for *Petromyzon marinus* L. [see Barreiro-Iglesias et al. (2008b) for a table of equivalences with the days of development]. Embryos and prolarvae were fixed in 4% paraformaldehyde in phosphate-buffered saline (PBS) during 24 hours at 4ºC, dehydrated in increasing methanol concentrations and stored in 100% methanol at -20ºC until the day of use. All experiments conformed to European and Spanish guidelines on animal care and experimentation and were approved by the Bioethics Committee at the University of Santiago de Compostela.

*Generation of a sea lamprey RGMA riboprobe*

The *RGMA* riboprobe was transcribed from a cDNA sequence of 581 nucleotides (GenBank accession number: EU449948.1) previously cloned in pGEM-T Easy vectors (Promega; Madison, WI, USA) (Shifman et al., 2009) . This 581-nucleotide sequence corresponds to a fragment of the sea lamprey *RGMA* transcript (NCBI Reference Sequence: XM_032973959.1). Digoxigenin (DIG)-labelled antisense riboprobes were synthesized using the linearized plasmids as templates and following standard protocols using a T7 polymerase (Nzytech, Lisbon, Portugal). Sense RNA probes were also previously generated as controls (Shifman et al., 2009) .

*Whole mount ISH and sectioning of embryos and prolarvae*

Whole mount ISH with the antisense riboprobe was performed using standard protocols, with the following adaptations. A proteinase K treatment (10 mg/ml) was performed for 30 minutes at 37ºC following embryo/prolarva rehydration. Riboprobes were incubated with the samples at 70ºC overnight in a concentration of 7.5 µl/ml in hybridization mix. An RNAse treatment step (37ºC for 30 minutes) was added to the post-hybridization washes. The sheep anti-DIG antibody conjugated to alkaline phosphatase (1:2000; Roche, Mannheim, Germany) was incubated over night at 4ºC in blocking buffer solution. Staining was conducted in BM Purple (Roche) at 37°C until the signal was clearly visible. No staining was detected when using sense probes as controls (Shifman et al., 2009) .

After whole mount ISH, lamprey embryos and prolarvae were cryoprotected with 30% sucrose in PBS, embedded in Tissue Tek (Sakura, Torrance, CA), frozen in liquid nitrogen-cooled isopentane, and cut serially on a cryostat (14 to 40 µm thick) in transverse or sagittal planes. Sections were dehydrated and mounted on subbed glass slides. Sections were photographed with a DP71 Olympus colour digital camera in a BX51 Olympus photomicroscope. Images were imported into Adobe Photoshop 2022 (Adobe Systems, Inc., San Jose, CA) and minimally adjusted for brightness and contrast, and labels were added. The schematic drawing was also generated with Adobe Photoshop 2022.

## References

[R1] Barreiro-Iglesias Anton, Gomez-Lopez Maria Pilar, Anadon Ramon, Rodicio Maria Celina (2010). Early Development of the Cranial Nerves in a Primitive Vertebrate, the Sea Lamprey, Petromyzon Marinus L.~!2008-08-19~!2008-09-30~!2008-10-24~!. The Open Zoology Journal.

[R2] Barreiro-Iglesias A, Villar-Cheda B, Abalo XM, Anadón R, Rodicio MC (2007). The early scaffold of axon tracts in the brain of a primitive vertebrate, the sea lamprey.. Brain Res Bull.

[R3] Barreiro-Iglesias A, Zhang G, Selzer ME, Shifman MI (2014). Complete spinal cord injury and brain dissection protocol for subsequent wholemount in situ hybridization in larval sea lamprey.. J Vis Exp.

[R4] Chen J, Laramore C, Shifman MI (2016). The expression of chemorepulsive guidance receptors and the regenerative abilities of spinal-projecting neurons after spinal cord injury.. Neuroscience.

[R5] Chen J, Shifman MI (2019). Inhibition of neogenin promotes neuronal survival and improved behavior recovery after spinal cord injury.. Neuroscience.

[R6] Cornide-Petronio ME, Barreiro-Iglesias A, Anadón R, Rodicio MC (2011). Retinotopy of visual projections to the optic tectum and pretectum in larval sea lamprey.. Exp Eye Res.

[R7] De Vries M, Cooper HM (2008). Emerging roles for neogenin and its ligands in CNS development.. J Neurochem.

[R8] Gessert S, Maurus D, Kühl M (2008). Repulsive guidance molecule A (RGM A) and its receptor neogenin during neural and neural crest cell development of Xenopus laevis.. Biol Cell.

[R9] González-Llera L, Santos-Durán GN, Sobrido-Cameán D, Núñez-González C, Pérez-Fernández J, Barreiro-Iglesias A (2023). Spontaneous regeneration of cholecystokinergic reticulospinal axons after a complete spinal cord injury in sea lampreys.. Comput Struct Biotechnol J.

[R10] González-Llera L, Shifman MI, Barreiro-Iglesias A (2023). Neogenin expression in ependymo-radial glia of the larval sea lamprey Petromyzon marinus spinal cord.. MicroPubl Biol.

[R11] González-Llera L, Sobrido-Cameán D, Santos-Durán GN, Barreiro-Iglesias A (2022). Full regeneration of descending corticotropin-releasing hormone axons after a complete spinal cord injury in lampreys.. Comput Struct Biotechnol J.

[R12] Matsunaga E, Tauszig-Delamasure S, Monnier PP, Mueller BK, Strittmatter SM, Mehlen P, Chédotal A (2004). RGM and its receptor neogenin regulate neuronal survival.. Nat Cell Biol.

[R13] Meléndez-Ferro M, Villar-Cheda B, Abalo XM, Pérez-Costas E, Rodríguez-Muñoz R, Degrip WJ, Yáñez J, Rodicio MC, Anadón R (2002). Early development of the retina and pineal complex in the sea lamprey: comparative immunocytochemical study.. J Comp Neurol.

[R14] Niederkofler V, Salie R, Sigrist M, Arber S (2004). Repulsive guidance molecule (RGM) gene function is required for neural tube closure but not retinal topography in the mouse visual system.. J Neurosci.

[R15] Oldekamp J, Krämer N, Alvarez-Bolado G, Skutella T (2004). Expression pattern of the repulsive guidance molecules RGM A, B and C during mouse development.. Gene Expr Patterns.

[R16] Rajagopalan S, Deitinghoff L, Davis D, Conrad S, Skutella T, Chedotal A, Mueller BK, Strittmatter SM (2004). Neogenin mediates the action of repulsive guidance molecule.. Nat Cell Biol.

[R17] Schmidtmer J, Engelkamp D (2004). Isolation and expression pattern of three mouse homologues of chick Rgm.. Gene Expr Patterns.

[R18] Shifman MI, Yumul RE, Laramore C, Selzer ME (2009). Expression of the repulsive guidance molecule RGM and its receptor neogenin after spinal cord injury in sea lamprey.. Exp Neurol.

[R19] Siebold C, Yamashita T, Monnier PP, Mueller BK, Pasterkamp RJ (2016). RGMs: Structural Insights, Molecular Regulation, and Downstream Signaling.. Trends Cell Biol.

[R20] Stahl B, Müller B, von Boxberg Y, Cox EC, Bonhoeffer F (1990). Biochemical characterization of a putative axonal guidance molecule of the chick visual system.. Neuron.

[R21] Villar-Cheda B, Abalo XM, Villar-Cerviño V, Barreiro-Iglesias A, Anadón R, Rodicio MC (2008). Late proliferation and photoreceptor differentiation in the transforming lamprey retina.. Brain Res.

[R22] Villar-Cheda B, Pérez-Costas E, Meléndez-Ferro M, Abalo XM, Rodríguez-Muñoz R, Anadón R, Rodicio MC (2006). Cell proliferation in the forebrain and midbrain of the sea lamprey.. J Comp Neurol.

[R23] Wilson NH, Key B (2006). Neogenin interacts with RGMa and netrin-1 to guide axons within the embryonic vertebrate forebrain.. Dev Biol.

